# Obtaining an Oily Ingredient Rich in PUFAS and Tocopherols and a High-Nutritional-Value Flour from Beans (*Phaseolus vulgaris* L.) by Supercritical CO_2_ Extraction

**DOI:** 10.3390/foods13010036

**Published:** 2023-12-21

**Authors:** Jesus Benites-Mena, Celia Vargas-De-La-Cruz, Claudia Vergara-Valdés, Jorge Jave-Nakayo, Jaime Ortiz-Viedma, Cielo Char, Marianela Inga-Guevara, Marcos Flores, Alberto Cepeda

**Affiliations:** 1 Departamento Académico de Farmacología, Facultad de Farmacia y Bioquímica, Bromatología y Toxicología, Universidad Nacional Mayor de San Marcos, Jirón Huanta 1182, Cercado de Lima, Lima 15001, Peru; jesus.benitesmena@unmsm.edu.pe (J.B.-M.); jorge.jave@unmsm.edu.pe (J.J.-N.); 2 Departamento de Ciencias Básicas, Facultad de Ciencias, Universidad Santo Tomás, Talca 3460000, Chile; claudiavergarava@santotomas.cl; 3 Departamento de Ciencia de los Alimentos y Tecnología Química, Facultad de Ciencias Químicas y Farmacéuticas, Universidad de Chile, Dr. Carlos Lorca Tobar 964, Santiago 8391063, Chile; cdchar@u.uchile.cl; 4 Facultad de Industrias Alimentarias, Universidad Nacional Agraria La Molina, Av. La Molina s/n, Lima 8380000, Peru; marianelainga@lamolina.edu.pe; 5 Departamento de Horticultura, Facultad de Ciencias Agrarias, Universidad de Talca, Talca 3460000, Chile; marcos.flores@utalca.cl; 6 Laboratorio de Higiene, Inspección y Control de Alimentos (LHICA), Departamento de Química, Analítica, Nutrición y Bromatología, Universidad de Santiago de Compostela, 27002 Lugo, Spain; alberto.cepeda@usc.es

**Keywords:** supercritical extraction, oil bean, flour bean, PUFAS, tocopherols, amino acids

## Abstract

The objective of this work was to carry out a preliminary study of the fractionation by supercritical CO_2_ (sc-CO_2_) extraction of two varieties of Peruvian beans (*Phaseolus vulgaris* L.), white (WB) and red (RB), to obtain two novel products: an oil rich in essential fatty acids and tocopherols and a defatted flour with high nutritional value and amino acids. The extraction temperature and pressure were optimized using the response surface methodology (RSM) and the extraction kinetics were optimized using the Spline equation. The results revealed that the best extraction conditions for WB and RB were 396.36 Bar, 40.46 °C, with an efficiency of 1.65%; and 391.995 Bar, 44.00 °C, with an efficiency of 1.12%, respectively. The WB and RB oils presented a high degree of polyunsaturation (63.2 and 52.8%, respectively), with oleic, linoleic, and linolenic fatty acids prevailing. Gamma-tocopherol was the predominant antioxidant in both oils. The residual flours (WB and RB) obtained after extraction with sc-CO_2_ had a high average content of proteins (23%), carbohydrates (61%), and minerals (3%). The limiting amino acids of WB were: Fen + Tyr, Leu, Lys, and in RB, only Leu was limiting. The viscosity of the solutions (20%) of the WB and RB flours mainly adjusted to the Waele’s Ostwald model (r = 0.988). It is concluded that both products (oil and bean flour) obtained in an optimized manner using an eco-friendly technology with sc-CO_2_ have high nutrient and bioactive component content and can be used in the development of new ingredients and healthy foods of plant origin.

## 1. Introduction

The bean *Pasheolus vulgaris L*. is a legume with worldwide distribution that represents half of the total legumes consumed in the form of grains [1]. Annual global production is estimated to be approximately 31 million tons, representing ~27% of the global production of major crops [1,2,3]. On the other hand, in various studies, it has been observed that climate change has produced heat waves and water stress, which lead to losses in legume crops, lower nutritional quality, and reduced bioactive compounds in beans, which leads to a residual biomass [4,5]. As a nutritional source, the bean is considered very valuable because it is an important source of protein, dietary fiber, and starch, as well as vitamins, minerals, and bioactive compounds such as tocopherols and phenols [1,6,7]. In addition, it has a good content of essential amino acids [8,9] and unsaturated fatty acids, such as linolenic acid and linoleic acid [9].

Notable differences in nutritional and antioxidant properties between bean species have suggested their potential for addressing type 2 diabetes [10]. In addition, bean leaves have beneficial properties for cardiovascular health [11,12]. However, one in seven consumers avoids the consumption of beans due to their heaviness and flatulence effect, which is due to the high content of fiber, galactooligosaccharides, and indigestible starch that tends to increase when the bean is cooked [13]. The excess of gas and water causes the intestinal wall to stretch and expand, which causes exaggerated feelings of pain and discomfort in people with irritable bowel syndrome [14]. On the other hand, beans in a dry state are not frequently consumed because they are unattractive to consumers, which leads to a loss of opportunity for the population to acquire their healthy effects through diet.

Therefore, it is necessary to implement innovative processes for the development of new food formats from beans. Currently, products that incorporate beans in new forms of functional ingredients, such as flours and pastas for use in the development of healthy foods, have proven to be a potential strategy to increase the utilization of dried beans [15]. One way to increase bean consumption is to break them down into their nutritional and bioactive compounds to diversify their incorporation in the form of new functional ingredients, such as essential oils, flours, and pastas, for use in the development of healthy foods, which has proven to be a potential strategy to increase the use of dried beans. Additionally, their residues and agricultural waste could be used for the development of new nutritional, easily digestible, and healthy foods and supplements. In this way, greater acceptability could be generated by physiologically sensitive consumers, such as people with celiac disease, allergies, and irritable colon, among others [15,16]. One of the methods to selectively extract bioactive compounds corresponds to the use of supercritical fluids with CO_2_ (sc-CO_2_) [17,18]. Thanks to its non-polar solvent property (sc-CO_2_), this technique constitutes a productive and clean alternative for the recovery of plant foods and their waste [17,19,20,21,22,23,24,25].

However, there are few studies that have used sc-CO_2_ extraction for the extraction of lipophilic components of beans or their residues that compare the composition of bean oil with oils obtained by traditional methods intended for the formulation of new ingredients [15]. Likewise, the nutritional value of the residue obtained after extraction with sc-CO_2_ has not been determined, nor have its physical properties in solution.

The objective of this work is to generate new food formats derived from beans by applying the extraction of non-polar compounds from white and red bean discards (WB and RB, respectively) using sc-CO_2_. The two new products correspond to an oil rich in essential fatty acids and tocopherols, and a defatted flour with high nutritional value in proteins, amino acids, and fiber. For this, the sc-CO_2_ extraction variables were optimized using a response surface experimental design (RSM) together with an adjustment of the extraction kinetics to the Spline model.

## 2. Materials and Methods

### 2.1. Beans Samples

Samples of white and red beans (WB and RB) called ñuñas were collected from the Ancahs area (Wari province, Peru; 9°32′ S 77°32′ W/−9.53, −77.53). The WB and RB samples were freeze-dried to a final humidity of 4.6 ± 1.2% (%w) and ground. The WB and RB flours obtained were sieved (586 ± 31 μm) and packed in polyethylene bags and then stored at −20 °C until analysis.

### 2.2. Obtaining Lipid Extract from Beans by Means of Supercritical CO_2_

Supercritical CO_2_ (sc-CO_2_) extraction of bean oils was integrated with an automatic extractor model Speed NP 7071 (Applied Separation Inc., Allentown, PA, USA) equipped with a pneumatic pump, chiller, compressor, column furnace, relief-purge valve, flow meter, and collection capillary. Samples (25 g) were mixed with Celite^®^ 545 (Merck KGaA 64271 Darmstadt, Germany) to enhance supercritical CO_2_ diffusion for oil extraction and then placed in a heavy-walled stainless steel cylindrical column. Some fiberglass was placed at both ends of the stripping column to prevent CO_2_ from carrying solids out of the system. After reaching the desired pressure and temperature, the extraction system was held for 15 min to allow sc-CO_2_ to adequately saturate the samples in the extraction column. The CO_2_ was allowed to flow at a rate of 1 mL/min. Then, all oil samples were placed into 25 mL vials and stored at −20 °C until needed for tocopherol and fatty acid profiling analysis. The yield of oil in beans (% by weight) was estimated by the relationship between the amount of oil extracted with sc-CO_2_ and the amount of dry matter.

#### 2.2.1. RSM Optimization of Oil Extraction by sc-CO_2_

The response surface methodology (RSM) was used using a central compound design (CCD) to determine the effect of two variables on the yield of bean oil and to identify the optimal conditions for its extraction. For the data analysis and the establishment of the model, the STATGRAPHICS plus 5.1 statistical and graphic analysis software was used. The coded pressure (X1, where P is the pressure in Bar) and the coded temperature (X2, where T is the temperature in °C) were chosen as independent variables, with three levels for each of them, for the extraction yield of oil (%). The parameters and levels were established based on previous studies [15], and a value of 45 °C was selected as the maximum temperature to preserve the integrity of the thermolabile compounds. The levels of independent variables and their levels for the CCD design were:

Coded pressure, X1 (Bar): 380, 400, 420, (levels: −1, 0, 1)
Coded temperature, X2 (°C): 35, 40, 45 (levels: −1, 0, 1)

A total of twelve experiments were carried out, composed of four factorial points, four extra points (star points), and four repetitions for the central point. Experiments were performed in randomized order to minimize the effects of uncontrolled actors. With these data, RSM were built considering the oil extraction yield (Y) as the response variable.

A second order model (type Y = β 0 + β 1 X 1 + β 2 X 2 +⋯+ β p X p) was used to describe the response variable Y as a function of the independent variables (X1 and X2).

#### 2.2.2. Modeling of the Extraction Kinetics with sc-CO_2_

The oil extraction curves with sc-CO_2_ were fitted to the Spline model described by Sovová (2012) [25]. From the option of the optimal operating conditions obtained by RSM, an extraction curve with sc-CO_2_ was made, which was modeled using the MATLAB R2020b software (California City, CA, USA). The model describes three consecutive stages defined by the rate of extraction and associated with the release mechanisms. The first stage corresponds to a constant rate (CER) described by convection, the second to a falling rate (FER) defined by convection and diffusion, and the third to a period controlled by diffusion (DC). Each extraction stage is described by straight lines represented by Equations (1)–(3).

Y = b0 + b1 × t for t < t_CER_ (rate constant: Convection) (1)

Y= b0 − t_CER_ × b2 + (b1 + b2) × t for tcer < t< t_FER_ (Convection-diffusion) (2)

Y= b0 − t_CER_ × b2 – t_FER_ × b3 + (b1 + b2 + b3) × t for t_FER_ < t (DC Diffusion) (3)

where, Y corresponds to the oil extraction yield per sc-CO_2_; bi (i = 0, 1–3) are the linear coefficients of each stage; t_CER_ is the time period _CER_; and t_FER_ is the FER time period.

### 2.3. Characterization of Lipids Obtained by sc-CO_2_

#### 2.3.1. Fatty Acid and Tocopherol Analysis

The fatty acid composition of the oil obtained by extraction with sc-CO_2_ was determined by gas chromatography according to the official method Ce 2-66 [26] using an HP-5890 gas chromatograph (Hewlett-Packard, Palo Alto, CA, USA) with a 50 m long bpx-70 fused silica column, 0.25 µm film thickness, and 0.25 mm internal diameter; along with an Fid detector and a split injection system calibrated at 90:10. The fatty acid methyl esters (FAMEs) obtained from Sigma-Aldrich (St. Louis, MO, USA) were prepared as follows: 100 mg of oil was mixed with 5 mL of 0.5 N sodium hydroxide solution in methanol and held in a thermoregulated bath for 5 min at 100 °C. Then, 5 mL of 12.5% boron trifluoride in methanol was added and heated for 3 min. Finally, 1.5 mL of petroleum ether and saturated sodium chloride solution were added. After gently shaking, the mixture was allowed to stand in order to promote phase separation to extract the FAME dissolved in petroleum ether.

#### 2.3.2. Tocopherol Content

Tocopherol contents of the oil obtained by extraction with sc-CO_2_, were determined using a High Performance Liquid Chromatography (HPLC) system according to the standard method AOCS Ce 8-89 [27] LiChro-CART Superspher Si 60 column (ID 250 × 4 mm, particle size 5 µm; Merck, Darmstadt, Germany) was used. The mobile phase was propan-2-ol in hexane (1.0/99.0, *v*/*v*) at a flow rate of 1 mL/min. The HPLC system consisted of a Merck-Hitachi L-6200A pump (Merck, Darmstadt, Germany), a Rheodyne 7725i injector with a 20 μL sample loop, a Hitachi Chromaster 5440 fluorescence detector, and a Merck-Hitachi D-chromato-integrator 2500. The excitation and emission wavelengths were in the ranges of 290 nm and 330 nm, respectively. Tocopherols were identified using Calbiochem standards (Merck, Darmstadt, Germany) as external standards.

### 2.4. Chemical and Physical Analysis Post Extraction with sc-CO_2_

#### 2.4.1. Nutritional Analysis

After extracting the lipid fraction with supercritical CO_2_, the residual defatted flour was subjected to a nutritional analysis according to the official methods [28]. The moisture and ash content were extended by gravimetric methods, the proteins by the Kjeldahl method, and the lipids by the Soxhlet method with petroleum ether. The carbohydrates were determined by the Antrona colorimetric method after digestion with analytical grade perchloric acid. The colored complex formed between the anthrone and the soluble sugars resulting from starch hydrolysis was read at 760 nm and expressed as g glucose/100 g [20].

#### 2.4.2. Amino Acid Analysis

The amino acids were determined using high-performance liquid chromatography (HPLC) according to the method of Alaiz et al. (1992) [29]. A sample equivalent to 2 mg of protein was weighted in a hydrolysis tube and then 4 mL of 6.0 M hydrochloric acid was added. D, L-a-aminobutyric acid was used as the internal standard. The solution was gassed with nitrogen and sealed, then it was incubated in an oven at 110 °C for 24 h. The amino acid hydrolizate was dried in a Büchi Rotavapor (Büchi Labortechnik, meierseggstrasse, Switzerland) and the amino acids were dissolved in 25 mL of borate buffer (1 M, pH 9.0). An amount totaling 5 mL of this solution was derivatized with 4 μL of diethyl ethoxymethylene-malonate at 50 °C for 50 min with vigorous shaking. A total of 20 μL of this derivatized solution was injected directly into the HPLC Merck-Hitachi L-6200A pump (Merck, Darmstadt, Germany). The separation of derivatives was obtained using a 300 × 3.9 mm i.d. reversed-phase column Nova-Pack C18; particle size, 4 lm (Waters, Milford, MA, USA). Detection was accomplished using a Model L-4250 UV-vis detector (Merck-Hitachi) at 280 nm. The solvents used were: (A) 25 mM sodium acetate containing 0.02% sodium azide (pH 6.0) and (B) acetonitrile. The solvent was delivered to the column at a flow rate of 0.9 mL/min as follows: time, 0.0–3.0 min, linear gradient from A-B (92:8) to A-B (88:12); 3.0–6.0 min, linear gradient from A-B (88:12) to A-B (86:14); 6.0–13.0 min, elution with A-B (86:14); 13.0–22.0 min, linear gradient from A-B (86:14) to (79:21); 22.0–35.0 min, linear gradient A-B (79:21) to A-B (69:31). For the determination of tryptophan, a rapid and simple acid ninhydrin method described by [30] and adapted for the colorimetric determination of tryptophan in protein extracts of beans by [31] was used.

### 2.5. Physical Properties

#### Flow Properties of Bean Flour

For the analysis of the flow properties of bean flours, 10% (*w*/*v*) flour solutions of WB and RB were prepared, which were heated to 80 ± 0.5 °C and analyzed by controlling the temperature of the solution using a water bath (HAAKE SC 100). Shear stress and shear rate data were collected as flow curves using a rheometer (HAAKE RheoStress 1, Thermo Fisher Scientific, Karlsruhe, Germany) connected to a software-controlled interface (HAAKE RheoWin, Data Manager software package version 4.30. 0016). The experiment was carried out using concentric cylinder geometry (Z34 DIN Ti) with a gap of 0.8 mm. In each solution, four intervals ranging from 0.001 to 10 1/s were applied, increasing and decreasing using a linear ramp. The flow curves were analyzed and fitted using the χ^2^ method to the most optimal rheological model using HAAKE Rheowin software 4.91.0021. Flow curves using the Chi squared (χ^2^) method were fitted to the Herschel–Bulkley, Cross–Willianson, Casson, Ostwald de Waele (Power law), and Bingham models according to:

(4) $$\mathrm{Cross}–\mathrm{Willianson}:\;\tau =Ῡ\left(\frac{\eta_{\infty }+(\eta_{0}-\eta_{ꝏ})}{\left(1+{\left(\frac{Ῡ}{\gamma_{b}}\right)}^{n}\right)}\right)$$

(5) $$Herschel–Bulkley:\;\eta =\frac{\tau_{0}}{Ῡ+K^{*}{Ῡ}^{\left(n-1\right)}}$$

(6) $$Ostwald\;de\;Waele:\eta =Ῡ+K^{*}{Ῡ}^{\left(n-1\right)}$$

(7) $$Bingham:\;\eta =\frac{\eta_{p}+\eta_{0}}{Ῡ}$$

where, (γ) is the shear rate, *η* is the flow index, *K* is the consistency index, to is the yield stress, and *n* is the flow behavior index.

### 2.6. Statistical Analysis

Results are expressed as means with standard deviation. For the response surface analysis, the analysis of variance (ANOVA) was considered with a confidence level of 95%, using the STATGRAPHICS plus 5.1 statistical and graphic analysis software.

## 3. Results and Discussion

### 3.1. Oil Extraction Yield Optimization

**Figure 1:**
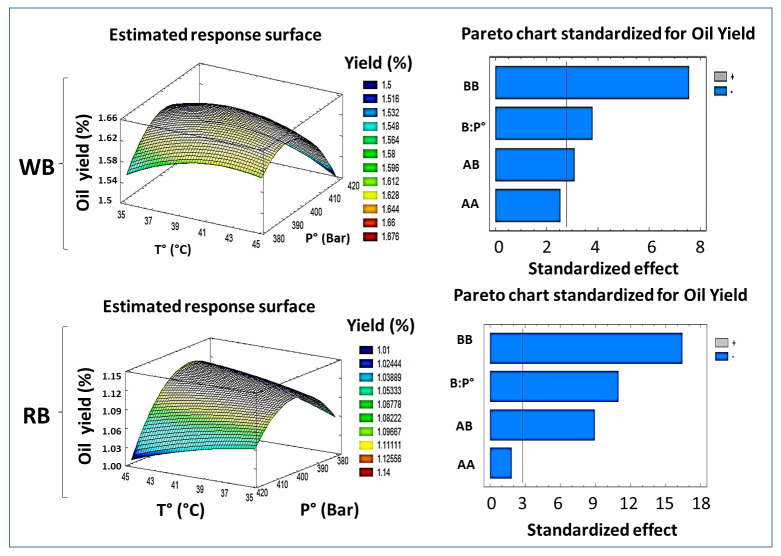
Estimated response surface and Pareto diagram for yield of WB and RB (A; Temperature, B; Pressure; *p* < 0.05).

Based on the CCD and the polynomial response equation obtained for the global extraction yield, the pressure had a significant effect on the linear and quadratic terms (*p* < 0.05) and therefore, a greater influence on the yield of oil extraction in both species. Therefore, the pressure as a first order variable is positive. Thus, increasing it would also increase the oil yield. This is confirmed with the Pareto diagram for the species WB and RB (Figure 1). Extraction yields increased with increasing pressure, as an increase in extraction pressure leads to a higher fluid density, the solubility of analytes increases [32]. Jokić et al. (2012) reported a similar finding regarding the supercritical fluid extraction rate of soybean oil, where yield increased with increasing extraction pressure [33]. The same results were reported by Duba and Fiori (2015) [34] regarding the extraction of grape seed oil. However, it is understood that any increase in pressure contributes to an increase in energy consumption, which can have a significant economic impact.

On the other hand, the temperature in its linear and quadratic form was not significant (*p* > 0.05) for either species. It is known that with increasing temperature, the sc-CO_2_ density decreases, but solute solubility may increase because of higher solute vapor pressure [26]. However, in this case, in both species, the increase in temperature meant a decrease in oil yield. This shows that the effect of pressure on the increase in CO_2_ density prevails over the increase in vapor pressure with temperature [35]. However, since the P*T interaction was significant (*p* < 0.05) in both species, the effect of pressure cannot be analyzed in isolation.

The optimal values of the process factors for both species and the corresponding yield obtained based on the CCD for WB were P° = 396.362 Bar and T° = 40.456 °C, achieving a yield of Y = 1.656% dw. On the other hand, for RB, P° = 391.995 Bar, T° = 44.003 °C, and oil yield Y = 1.127% dw. The regression models obtained were used to calculate the response surface for each variable separately. The estimated response surface for extraction pressure and temperature versus extraction yield (%) and their related contours are shown in Figure 1. In both response surface graphs, the positive slope shows a positive effect of pressure on oil yield; however, in both species, when maintaining a temperature of 40 °C, bean oil yield decreased with the increase in pressure from 400 to 420 Bar. The same results were found by Martínez-Ávila et al. (2022) [15] in the process of optimizing oil extraction from *Phaseolus vulgaris* L. using sc-CO_2_.

### 3.2. Kinetic Modeling of sc-CO_2_ Extraction

For the supercritical extraction, an extraction curve was calculated to determine the optimal time for oil extraction. This was determined by measuring the oil content every 15 min. It must be noted that most of the oil was extracted in the first 15 min; therefore, the extraction rates increased steadily in a linear fashion, allowing the CO_2_ to be saturated with oil for a period of constant growth [36]. After that time, the increase in yield was reduced dramatically so as to eventually plateau, and remained constant for the rest of the extraction process.

The adjustment parameters were:

WB; tCER = 15.90, tFER = 70.00, b0 = 0.1, b1 = 0.07, b2 = −0.06, b3 = −0.003 (r = 0.99)
RB; tCER = 1.80, tFER = 12.99, b0 = 0.01, b1 = 0.42, b2 = −0.35, b3 = −0.007 (r = 0.99)

**Figure 2:**
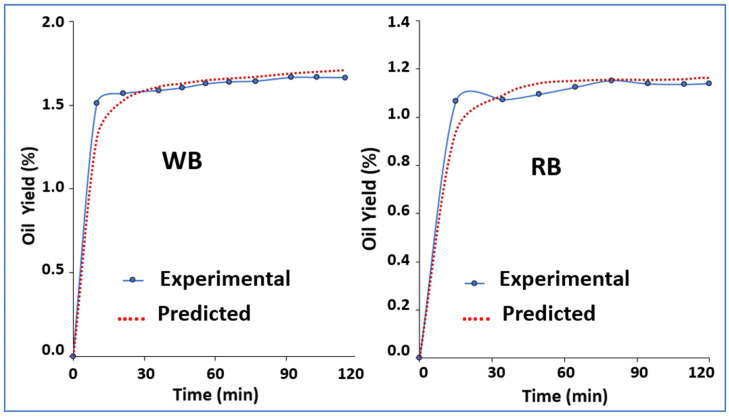
Oil extraction curves under optimal conditions (40 °C and 400 bar) obtained by supercritical extraction and its kinetic modeling using the Spline equation.

The adjustment to the Spline model (Figure 2) of the oil extraction from the WB and RB samples presented an adequate adjustment of the model to experimental data in both samples, as indicated by the error values and (r = 0.99). The Spline model consists of three stages (CER, FER, and DC). The CER stage is described by convective phenomena and is carried out at a constant speed; in the FER stage, the phenomena include convection and diffusion with decreasing speed; while the DC stage is controlled by diffusion phenomena [37]. The t_CER_ and t_FER_ values obtained in the RB sample were higher than those reported by Chañi-Paucar et al. (2022) [38] for branca sucupira (*Pterodon pubescens*) seeds and by Dos Santos et al. (2016) [39] for cumbaru oil (*Dipteryx alata*), while those obtained for the WB sample were similar to those reported by these same authors (3.29 min and 13.51 min). It is important to highlight that for the WB sample, these times are considered more interesting at the industrial level, since, as they are shorter, they imply a process that allows for higher productivity and lower costs. Regarding the parameters found, b1 indicates the rate of mass extraction in the CER stage. The values found in the WB and RB samples are lower than those reported by Fornereto Soldan et al. (2021) [40] for *Capsicum annuum*, which generally indicates a low extraction speed and yield for the samples analyzed in this study.

### 3.3. Composition of the Oil Extracted by sc-CO_2_

#### Fatty Acids and Tocopherols

Table 1 shows the profile of fatty acids and tocopherols for WB and RB oil, extracted with supercritical CO_2_. The results indicated that the RB oil presented a higher saturation than the WB, being the main fatty acid palmitic acid (C16:0). Likewise, both grains present a similar content in their content of monounsaturated fatty acids, with oleic acid (C18:1-ω9) being prevalent in WB and RB. On the other hand, WB oil has a greater polyunsaturated character than RB oil, with linoleic acid C18:2 ω9c and linolenic acid (C18:3 ω9) being the one with the highest content in both grains. Comparison of the lipid profile with that of other bean varieties (Table 1) indicates that the saturated fatty acid content of WB and RB is similar to that of other varieties such as kidney beans and pinto beans [41].

On the other hand, the level of monounsaturation of WB and RB, given mainly by the oleic acid content, is significantly higher than that of the black, kidney, great, and pinto bean varieties and only surpassed by soybean. However, the level of polyunsaturated fatty acids, provided mainly by linoleic and linolenic acids, is slightly lower than that of most bean varieties in Table 1, and the linoleic acid content of WB and RB is also lower than that of the soy bean. However, both beans have higher linolenic acid content. In a study [42], mean values of approximately 6% monounsaturated fatty acids for three freeze-dried raw varieties of common bean and 78% polyunsaturated fatty acids were found, being higher than the value found in WB and RB flours reported by Sutivisedsak et al. (2011) and Grelaap and Giinterb (1995) [41,43]. However, these values are similar to those reported for WB. On the other hand, in lentil cultivars, [44] determined that linoleic acid was the dominant fatty acid (40.73−47.06%), followed by oleic (20.11–28.00%), palmitic (12.67−14.82%), and alpha-linolenic (9.00–13.28%). On the other hand, it has been found that red kidney beans are a rich source of polyunsaturated fatty acids (71.1%), while white beans would have high amounts of saturated fatty acids (28.7%). However, due to the linolenic acid content reported for other bean and lentil varieties, it is important to highlight that WB and RB also constitute an important source of polyunsaturated acids.

From a structural point of view, it has been determined that in the structure of these lipids of plant origin, saturated fatty acids, such as stearic and palmitic acids, mainly occupy the *sn*-1 or *sn*-3 position, while unsaturated fatty acids, such as linoleic and linolenic acid, are mainly concentrated at the *sn*-2 position of the triacylglycerol molecule, while oleic acid is evenly distributed at the *sn*-1, *sn*-2, and *sn*-3 positions [45]. However, due to the linolenic acid content reported for other bean and lentil varieties, it is important to highlight that WB and RB also constitute an important source of polyunsaturated acids.

The native varieties of *Phaseolus vulgaris* L. WB and RB contained mainly γ-tocopherols as the dominant form (19.50 ppm and 12.20 ppm, respectively), followed by small amounts of δ-tocopherols, with total tocopherols of 19.82 ppm in WB and 12.47 ppm in RB. In addition, α- and β-tocopherols were also found, but in very low concentrations, and outside the quantification ranges of the methodology used. The results obtained here are lower than the high ranges reported for γ-tocopherol and total tocopherols determined by Sutivisedsak et al. (2011) [41] for black bean, kidney bean, great northern, and pinto bean. However, the tocopherol content is similar to that determined by Kan et al. (2018) [9] and Padhi et al. (2017) [46] who, respectively, reported 0.9–1.3 ppm and 22–35 ppm of total tocopherols on a dry basis for different samples of *Phaseolus vulgaris* L.

### 3.4. Chemical Analysis of Residual Bean Flour Post Extraction with sc-CO_2_

#### 3.4.1. Nutritional Analysis

**Figure 3:**
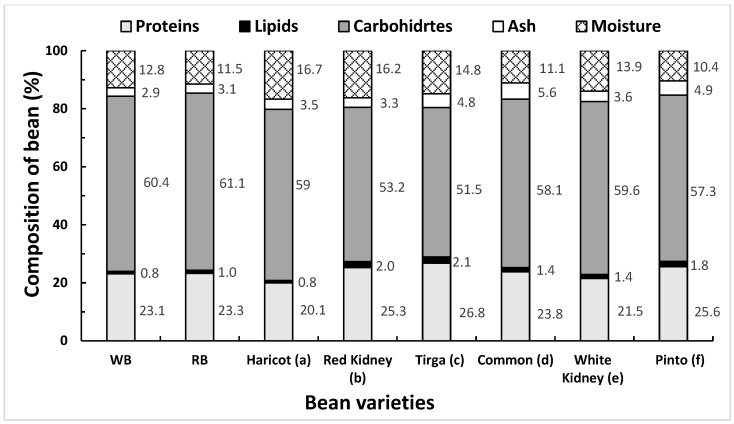
Nutritional composition of WB and RB and their comparison with the nutritional value of other varieties of beans. (**a**) [47], (**b**) [48], (**c**) [49], (**d**) [50], (**e**) [51], (**f**) [49].

Figure 3 shows the results of the proximal composition of the residual WB and RB samples analyzed after sc-CO_2_ extraction. The fat contents of WB and RB before sc-CO_2_ extraction were 1.4% and 2.1%, respectively. This initial lipid content in WB and RB is within that reported for other bean varieties, which fluctuates between 0.8 and 2.1% [9]. In general, the fat content of beans is around 2%, being made up mainly of phospholipids and triacylglycerols and minor amounts of diacylglycerols, hydrocarbons, stearyl esters, and hydrocarbons. These lipids can also be as phosphatidylcholine, phosphatidylethanolamine, and phosphatidylinositol [45]. In addition, fatty acids such as palmitic, oleic, and linoleic acids are also found.

The composition analysis for both beans indicated that the extraction by means of sc-CO_2_ had oil extraction yields of 57% and 47.6%, decreasing the oil content in the residues in WB and RB to 0.8% and 1.1%, respectively. On the other hand, the content and values of the other macronutrients are similar to those reported in other studies for *Phaseolus vulgaris* L; for example, moisture in the range of 10.4–16.7%, protein 20.1–26.8%; ashes 2.9–5.6%; and carbohydrates in the range of 51.5–61.1% [9,48,49,50,51]. Similarly, [52] reported moisture information in a range of 8.6–9.6 g/100 g, protein 18.8–21.4 g/100 g, fat 1.7–2.9 g/100 g, ash 3.6–3.8 g/100 g, and carbohydrates 64.3–67.1 g/100 g. In general, in *Phaseolus vulgaris* L., the protein range expands to between 18 and 30% of the dry weight, with albumins, glutelins, phaseolin, and prolamin being the most abundant.

On the other hand, from the protein value point of view, beans are a food with a great contribution of dietary protein for human nutrition. In addition, to avoid essential amino acid deficiency, they can be supplemented with other protein sources, such as wheat and other cereals [53]. The protein content of beans is similar to that of meat, ranging between 20 and 30% (Figure 3) Therefore, they are very appropriate to use as an ingredient in the preparation of hamburgers or other protein-based foods of plant origin. Bean proteins are poorly digestible, being very resistant to proteolysis in the human digestive tract, which is not a problem if bean flour is used in the formulation of foods that need to be processed, thus improving their digestibility and amino acid bioavailability [54].

#### 3.4.2. Amino Acids Analysis

##### Total Amino Acids

The WB and RB flours presented (Table 2) close values in the total amino acid content compared to the values found by Martino et al. (2012) [55] in the raw bean of the Perola variety. Even so, they have approximately 6% more total amino acids than those found in raw beans of the Carioca variety. Additionally, as observed by Martino et al. (2012), the total amino acids for the processed beans ranged from 11.89% to 14.05% (Talisman). Therefore, based on amino acid percentage, only raw beans are comparable with the values found in WB and RB flours. In a study reported by Kan et al. (2018) [9], it was found that the total amino acids of the legumes studied ranged from 14% (pea grown in Nanchang) to 36.04% (black soybeans grown in Jiazhuang). Soybean and black soybean had higher amino acids (26.93–29.52% and 31.21–36.04%, respectively) than the other legume samples (14.00–22.22%). Table 3 shows the amino acid composition of WB and RB, and their comparison with raw and cooked beans from the literature [55].

##### Essential Amino Acids

The RB flour, on average, presented a higher content of essential amino acids than the WB flour (15,547 and 11,794 g/100 g, respectively). On the other hand, both flours presented a higher value of essential amino acids than those of raw and cooked beans (9.12 and 6.58 g/100 g, respectively), where the values ranged between 4.76 and 6.34 g/100 g, respectively [55], with RB being the one that presented the highest content of lysine and total essential amino acids compared to WB.

##### Comparison with the FAO Standard

When comparing the amino acid content of WB and RB with the FAO/WHO amino acid scoring pattern (1981) for children from 2 to 5 years old (Table 2), the essential amino acids that presented a limiting concentration in WB and RB flours were leucine, phenylalanine + tyrosine, and tryptophan. The rest of the amino acids obtained a score greater than 1. The RB flour presented a higher content of amino acids that exceeded the FAO (1981) reference standard compared to the WB flour. On the other hand, lysine, which is usually the most deficient amino acid in cereals or legumes (FAO, Rome, Italy, 1981), was not limiting in WB and RB, while values of up to 1.78% in raw beans and 1.05% in cooked have been reported.

On the other hand, a comparison of the amino acid composition of beans consumed in Brazil in a raw state, as well as cooked (Table 2), shows that both WB and RB have higher or similar amounts of total essential amino acids, but with a lower lysine content.

Kan et al. (2018) indicated that the lysine content in legumes ranged from 0.90% (lentils grown in Mizhi) to 2.23% (black soybeans grown in Jiazhuang), while the sulfur amino acids, such as methionine, in the legumes analyzed were relatively low (0.03–0.28%) [9]. In line with the above, Mbithi-Mwikya et al. (2000) indicated that red beans contained 45.1% amino acids, presenting a very good relationship between essential and total amino acids, but were limited in combined methionine and cysteine, which was not observed in cereals such as millet [56,57].

##### Non-Essential Amino Acids

The WB and RB raw bean flours (Table 3), on average, presented similar values in non-essential amino acid content (7222 and 5616, respectively) to those found by Martino et al. (2012) [55] in processed beans. However, this is not the case for raw beans, which presented non-essential amino acid totals of 14.11 (Perola) and 10.55 (Carioca); that is, at least double that the content found in flours.

### 3.5. Physical Properties of Residual Bean Flours

#### Residual Bean Flow Properties

**Figure 4:**
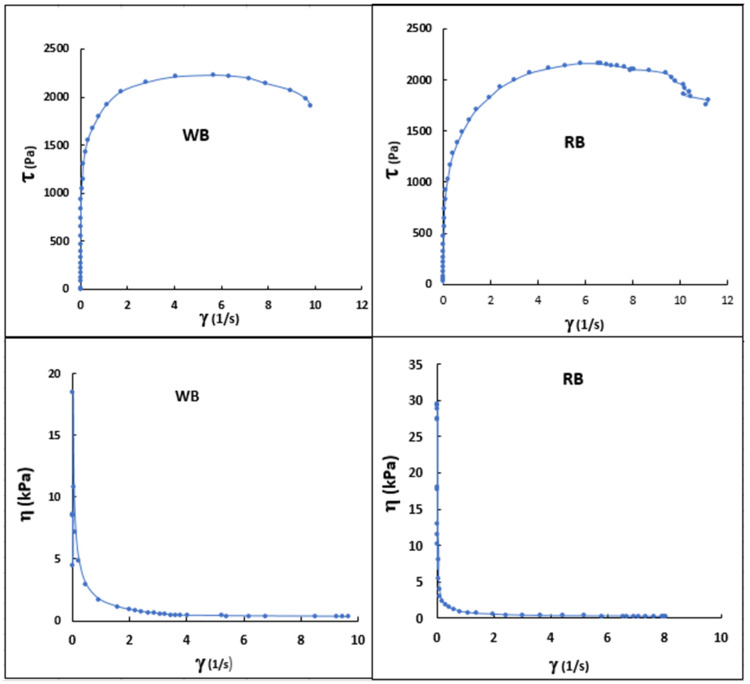
Flow properties of WB and RB solutions (25% *w*/*v*). τ vs. γ (top graphics) and variation of η as a function of γ (lower graphics) of the WB and RB solution.

Figure 4 (top graphics) show the flow curves (τ vs. γ) of 10% aqueous suspensions of WB and RB flours, both previously heated to 90 °C. It is observed that η tends to increase proportionally with the increase of γ until reaching a maximum limit. This indicates that up to this cut-off point, the structure of the WB and RB solution increased in viscosity, offering greater resistance to deformation. The increase in viscosity (top graphics) in flour WB and RB would be due to the swelling and gelatinization of starch, a biopolymer that is normally found in values close to 40% of the composition of the beans. Likewise, the increase in viscosity would be complemented by the gelling of the endogenous proteins of the beans since, like starch, they are found in a high content in the legume, ranging from 18 to 26%. Subsequently, at greater values of η, γ tended to decrease, indicating that the structures of the semi-gel formed were breaking or thinning as greater shear was applied. This behavior was similar for both WB and RB suspensions.

The above behavior (Figure 4) indicates that the flow properties of WB and RB were intensified due to the respective damage to the structure of the gelled biopolymeric components (starch, pectin, proteins, etc.), which is typical of pseudoplastic behavior in the analyzed range.

The pseudoplastic behavior of WB and RB is similar to that of other homogenized foods, such as strawberry, apricot, peach, plum, raspberry, guava, mango, and pineapple, and in the pulps of various foods, such as mango and pineapple [58,59,60]. The sudden increase and subsequent rapid drop in viscosity that the WB and RB soups presented should be due to the swelling and subsequent rupture of the starch grain.

Table 3 presents the representative rheological models that best fit the behavior of η vs. γ ˙ of the WB and RB suspensions. The results indicated that for WB and RB, the model that best fit was the Ostwald de Waele model (r = 0.936 and 0.988) followed by Cross–Willianson (r = 0.892 and r = 0.988) and Herschel–Bulkley (r = 0.855 and r =0.882), respectively. Since η is a measure of the non-Newtonianity of the fluid, the results indicate that the further from unity the value of η , the more non-Newtonian the behavior of the fluid. We see that for the Cross–Willianson model, the WB solution represents a moderately more representative behavior of a non-Newtonian fluid than RB. On the other hand, a higher parameter K indicates an increase in the concentration of solids, making the fluid more viscous. In other investigations related to the rheological behavior in green plantain flour paste [61], it was determined that the modeling that best fits this type of paste is the Herschel–Bulkley model.

**Table 3:** Representative rheological models that best fit the behavior of η v/s γ ˙ of WB and RB solutions.

Rheological Model	Coefficients	Bean Flour Suspensions (10%)	
		WB	RB
*Cross–Willianson*	η _o_(10^7^)	3.3 ± 0.3	2.1 ± 0.0
	η _ꝏ_	35.86 ± 2.8	3442 ± 67
	γ_o_ (10^−2^)	5.2 ± 0.0	4.8 ± 0.1
	η (10^−2^)	95 ± 2	91 ± 1
	**r**	0.892	0.980
*Herschel–Bulkley*	**τ_0_**	317.9 ± 21.2	−177.6 ± 0.0
	**K**	453 ± 33	1588 ± 87
	η (10^−2^)	5.2 ± 0.1	22.1 ± 0.1
	**r**	0.855	0.882
*Ostwald de Waele*	**K**	129 ± 8.1	1480 ± 97
	η (10^−2^)	21.0 ± 0.1	29.1 ± 0.0
	**r**	0.936	0.988
*Bingham*	γ	81.7 ± 5.6	500.4 ± 33.4
	η	21.2 ± 1.1	504.2 ± 36.2
	**r**	0.7173	0.833

For each model, the rheological variables (τ0, K, η) obtained correspond to the mean ± standard deviations (n = 3) of WB and RB; r: Coefficient of determination; τ0: yield stress; K: consistency index; η: flow index.

## 4. Conclusions

Optimization of the response surface model revealed that pressure was the variable with the greatest effect on oil extraction performance for WB and RB, with the optimum being 40 °C. Only in RB did temperature have a positive effect when combined with pressure. The lipid extraction kinetics fit well to the Spline equation, indicating that the extraction in WB was more effective than in RB. The WB and RB oils presented a high degree of polyunsaturation (63.2 and 52.8%, respectively), with oleic, linoleic, and linolenic fatty acids prevailing. Gamma-tocopherol was the predominant antioxidant in both oils. The residual flours (WB and RB) after sc-CO_2_ extraction had a high average content of proteins (23%), carbohydrates (61%), and minerals (3%). The limiting amino acids of WB were mainly Fen + Tyr, Leu, and Lys, and in RB, only Leu was limiting. The flow properties of the solutions (20%) of the WB and RB flours mainly fit Waele’s Ostwald model (r = 0.988). It is concluded that both products (bean oil and flour), obtained in an optimized way through an ecological technology with sc-CO_2_, have a high content of nutrients and bioactive components that can be used in the development of new ingredients and healthy foods of plant origin. In the future, the application of combined extraction methods (supercritical and enzymatic, etc.) that would allow the maximum nutritional and bioactive components of beans to be individually obtained is expected.

## Figures and Tables

**Table 1:** Fatty acid compositions (% of methyl esters) and Tocopherols (ppm) of crude oils from beans (WB, RB), black bean, kidney bean, great northern bean, and pinto bean (oils shown for comparison *).

Fatty Acid (%)	WB	RB	Black Bean	Kidney Bean	Great Northern	Pinto Bean
C14:0	0.2 ± 0.0	0.1 ± 0.0	0.1	0.1	0.1	0.1
C16:0	13.2 ± 1.1 a	18.2 ± 3.4 b	10.7	12.3	11.5	12.7
C18:0	2.4 ± 0.2 a	6.0 ± 1.4 b	1.8	1.4	2.0	1.7
C20:0	0.5 ± 0.0 a	1.6 ± 0.3 b	0.5	0.5	0.5	0.3
C22:0	1.2 ± 0.1	1.3 ± 0.1	0.5	0.7	0.5	0.4
Sat	17.5	27.2	13.8	15.0	14.6	15.2
C16:1 ω9	0.2 ± 0.0	_	0.3	0.3	0.2	0.2
C18:1 ω9	15.7 ± 2.1	15.7 ± 2.5	9.3	9.5	5.2	5.9
C18:1 ω11	2.1 ± 0.1 a	4.0 ± 0.8 b	1.9	2.6	1.8	1.7
C20:1 ω11	0.3 ± 0.0	_	0.2	0.2	0.1	0.1
Monounsat	18.3	19.7	11.7	12.6	7.3	7.9
C18:2ω9c	29.3 ± 2.7	24.8 ± 5.3	31.1	24.1	33.4	32.1
C18:3 ω9	33.6 ± 4.1	25.7 ± 4.1	41.7	46.0	42.8	43.3
C22:2	0.9 ± 0.1 a	2.3 ± 0.3 b	1.0	1.8	1.2	1.2
C22:3	0.4 ± 0.0	0.3 ± 0.0	0.7	0.5	0.7	0.3
Polyunsat	63.6	52.8	73.6	71.0	77.1	75.8
Tocopherols (ppm)						
α-Tocopherol	–^a^	–^a^	110 ± 4	151 ± 4	25 ± 2	29 ± 1
β-Tocopherol	–^a^	–^a^	–^a^	–^a^	–^a^	–^a^
γ-Tocopherol	19.50 ± 4.1	12.20 ± 2.4	2692 ± 21	2380 ± 19	2828 ± 24	2737 ± 32
δ-Tocopherol	3.2 ± 0.1	2.7 ± 0.2	157 ± 6	137 ± 3	116 ± 3	88 ± 3
Total Tocopherols	22.7	12.47	2959	2668	2969	2854

* Ref. [41]. Values represent the mean ± standard deviations. ^a^: Not quantified. Different lowercase letters mean significative difference between WB and RB samples (*p* < 0.05).

**Table 2:** Amino acid composition of WB and RB, and their comparison with raw and cooked beans. Comparison with the FAO reference protein.

	*Raw Beans*				*Cooked Beans*				FAO ref. *	*Raw Beans*	
Aminoacid	WB	RB	Perola	Carioca	Ouro Blanco	Diamante Negro	RBS Radiante	Talisman	Aminoacid Score	WB	RB
(g/100 g)											
**Essential amino** **acid**											
Phen + Tyr	3.328 ± 0.524 a	3.885 ± 0.099 b	1.29	0.94	1.19	1.57	1.42	1.62	6.3	5.3 *	6.2 ^+^
His	0.795 ± 0.011 a	1.041 ± 0.035 b	0.58	0.44	0.39	0.36	0.37	0.44	1.9	4.4 ^+^	5.5 ^+^
Isoleu	1.273 ± 0.132 a	1.436 ± 0.043 b	1.16	0.81	0.43	0.55	0.56	0.62	2.8	4.5 ^+^	5.1 ^+^
Leu	2.941 ± 0.328 a	3.378 ± 0.138 b	1.77	1.30	0.84	1.15	1.16	1.20	6.6	4.3 *	5.0 *
Lys	0.325 ± 0.001 a	0.379 ± 0.002 b	1.78	1.25	0.67	0.97	1.05	0.94	5.8	4.5 *	6.5 ^+^
Met + Cys	0.647 ± 0.008 a	0.728 ± 0.005 b	0.26 3	0.17	0.20	0.24	0.22	0.27	2.5	2.6 ^+^	2.9 ^+^
Threonine	1.665 ± 0.012 a	1.945 ± 0.114 b	0.98	0.75	0.48	0.59	0.51	0.53	3.4	3.9 ^+^	5.7 ^+^
Tryp	1.102 ± 0.029	1.101 ± 0.018	nd	nd.	nd	nd	Nd	nd	1.1	1.1	1.1
Val	1.485 ± 0.033 a	1.654 ± 0.289	1.30	0.91	0.56	0.64	0.64	0.72	3.5	4.2 ^+^	4.7 ^+^
Subtotal	11.794 ± 1.08 a	15.547 ± 3.06 b	9.12	6.58	4.76	6.07	5.93	6.34			
**Non** **essential amino** **acid**											
Ala	0.833 ± 0.013	0.821 ± 0.067	0.90	0.70	0.61	0.67	0.64	0.69			
Arg	2.101 ± 0.043 a	1.201 ± 0.088 b	2.06	1.60	0.80	0.79	0.7	0.93			
Asp. acid	1.678 ± 0.064 a	1.116 ± 0.982 b	3.80	2.77	1.70	1.75	1.66	1.78			
Glut. acid	2.221 ± 0.005	2.101 ± 0.873	4.29	3.15	2.15	2.12	1.99	2.26			
Gly	0.768 ± 0.041 a	0.511 ± 0.211 b	0.81	0.59	0.49	0.51	0.47	0.51			
Prol	0.838 ± 0.025 a	0.796 ± 0.013 b	0.90	0.70	0.62	0.65	0.63	0.72			
Ser	1.222 ± 0.055 a	1.198 ± 0.343 b	1.35	1.03	0.76	0.84	0.74	0.82			
Subtotal	7.222 ± 0.026 a	5.616 ± 0.998 b	14.11	10.55	7.13	7.33	6.83	7.71			
Total	23.222 ± 4.589	23.291 ± 4.598	23.22	17.13	11.89	13.40	12.76	14.05			

*: limiting amino acid, ^+^: amino acid that exceeded the FAO reference protein. Different lowercase letters mean significative difference between WB and RB samples (*p* < 0.05).

## Data Availability

The data presented in this study are available on request from the corresponding author.
